# Nusinersen in type 0 spinal muscular atrophy: should we treat?

**DOI:** 10.1002/acn3.51126

**Published:** 2020-11-04

**Authors:** Eloisa Tiberi, Simonetta Costa, Marika Pane, Francesca Priolo, Roberto de Sanctis, Domenico Romeo, Francesco D. Tiziano, Giorgio Conti, Giovanni Vento, Eugenio Mercuri

**Affiliations:** ^1^ Neonatology Unit Department of Woman and Child Health and Public Health Fondazione Policlinico Universitario Agostino Gemelli IRCCS Università Cattolica del Sacro Cuore Rome Italy; ^2^ Pediatric Neurology Department of Woman and Child Health and Public Health Fondazione Policlinico Universitario A. Gemelli IRCCS‐Università Cattolica del Sacro Cuore Rome Italy; ^3^ Centro Clinico Nemo Fondazione Policlinico Universitario Agostino Gemelli IRCCS Rome Italy; ^4^ Section of Genomic Medicine Department of Life Science and Public Health Università Cattolica del Sacro Cuore Rome Italy; ^5^ Pediatric Intensive Care Unit Fondazione Policlinico Universitario Agostino Gemelli IRCCS Rome Italy

## Abstract

A male infant affected by type 0 SMA with one copy of SMN2 received early treatment with Nusinersen at the age of 13 days. He showed mild motor improvement 2 months after treatment started but despite also showing some minimal respiratory improvement, required tracheostomy at the age of 4 months and had increasing cardiac and autonomic dysfunction leading to exitus at 5 months. Our findings, expanding the results available on Nusinersen, confirm its relative efficacy in the most severely affected infants and provide clinical evidence to be used at the time requests for treating severe infants are discussed.

## Introduction

Spinal muscular atrophy (SMA) is a neuromuscular disorder characterized by degeneration of spinal motor neuron. The clinical spectrum is broad, classically including three types with pediatric onset, with type 1 reported as the severe form of SMA.

A prenatal form, clinically more severe than type 1 was reported in 1999.^1^ This form, labeled as type 0 SMA, is characterized by a clear prenatal history of reduced fetal movements and is associated, at birth, with profound hypotonia and weakness, contractures, feeding difficulties, and severe respiratory insufficiency with need for resuscitation and mechanical ventilator support.^2^, ^3^

In infants with type 0 SMA the SMN2 copy number is often limited to one copy.^3^, ^4^

The advent of Nusinersen has changed the perspective of survival, motor, and respiratory function and quality of life in type 1 infants.^5^, ^6^, ^7^ We report our experience of a male infant affected by type 0 SMA who received early treatment with Nusinersen.

## Clinical Report

The index case is a male infant born by unrelated parents. There was a positive family history for SMA as the couple had a female child, diagnosed as type 0 SMA, who died at 23 days of life.

Amniocentesis performed at 20 weeks of gestation revealed homozygous deletion in the SMN1 gene, with one copy of SMN2 gene.

The mother reported reduced fetal movements throughout the pregnancy. The boy was born at 35 + 6 weeks of gestational age by caesarean section with a birth weight of 2470 gr (32° percentile), length of 45 cm (16° centile), and head circumference of 33.4 cm (77° percentile).

At birth, there was absence of spontaneous breathing requiring immediate tracheal intubation and mechanical ventilation.

Neurological examination showed marked generalized muscular weakness, profound generalized hypotonia, and tongue fasciculation and absent reflexes. There were feet, ankle, popliteal, and elbow contractures with hand deformities but no webbing. Suck and swallow reflexes were absent. Genetic tests at birth confirmed prenatal diagnosis.

**Figure 1:**
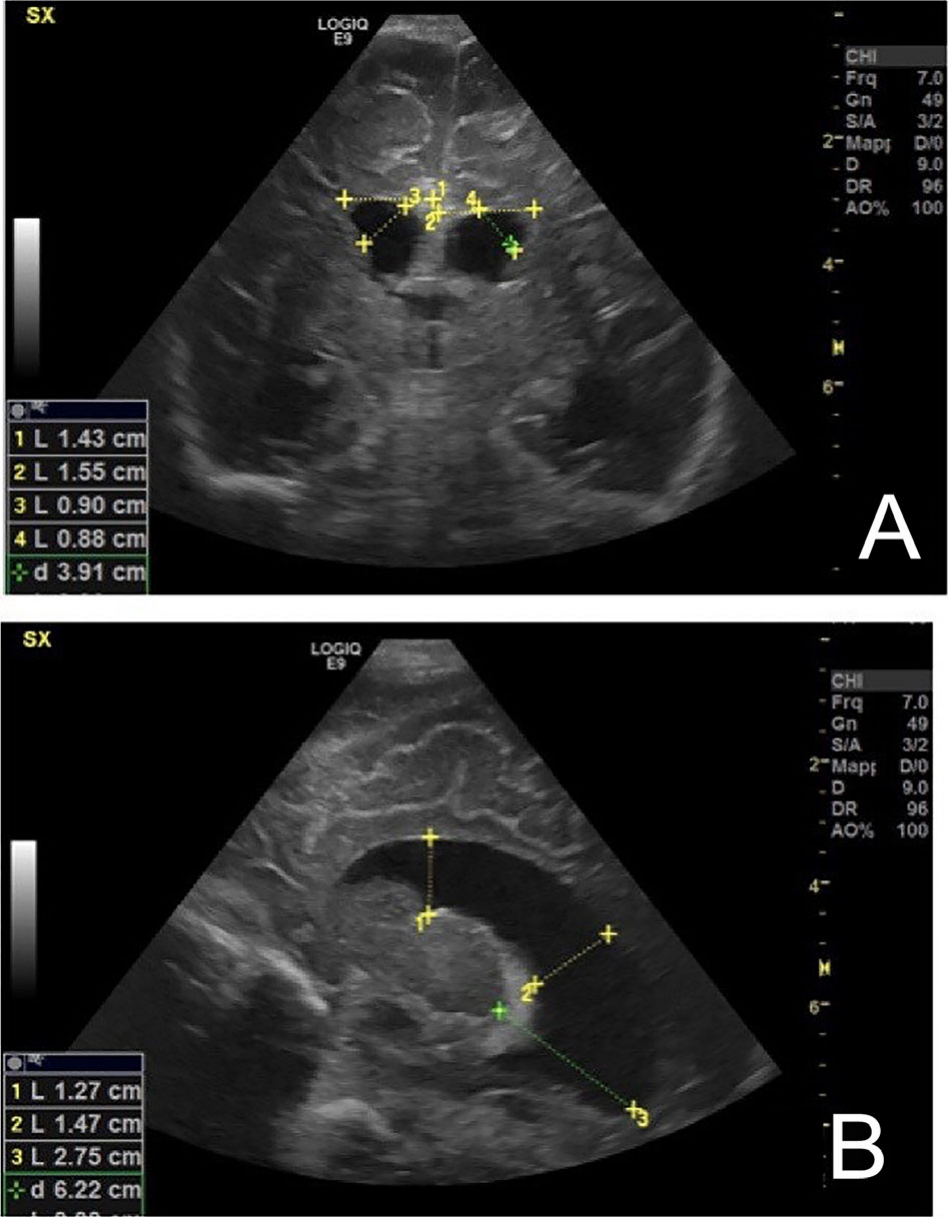
(A–B) Head ultrasound frontal and parasagittal sections at 3 month of life showing widening lateral ventricle. Right: Ventricular Index (VI) 1.43 cm, Anterior Horn Width (AHW) 0.90 cm, Ventricular Height (VH) 1.37 cm, Atrial Depht (AD) 1.95 cm; Talamo‐Occipital distance (TOD) 2.62 cm. No signs of hydrocephalus were detected.

Transthoracic echocardiography revealed two ostium secundum atrial sept defects and one apical sept defect. Cranial ultrasonography showed widening of ventricles but no signs of hydrocephalus and increased cisterna magna not associated with cerebellar hypoplasia (Fig. 1). The parents opted for a proactive approach, discussing the various treatment options and the possibility to perform tracheostomy and gastrostomy. They were informed of the lack of data on type 0 infants treated with Nusinersen but they felt that they would still consider Nusinersen treatment.

Before starting treatment, the child had a very poor respiratory drive and was on full time invasive respiratory support – synchronized intermittent mandatory ventilation. An attempt to reduce the level of ventilation by introducing a trial of continuous positive airway pressure (CPAP) of 6 cmH_2_O by endotracheal tube (ETT) failed as within 5 min from starting the EET CPAP trial the baby experienced severe hypoxia (SpO_2_ < 40%) and bradycardia (HR < 60 beats/min), requiring immediate restarting of mechanical ventilation. The transcutaneous monitor of blood gases showed TcPCO_2_ values of 75 mmHg, and TcO_2_ values of 40 mmHg. Hyperglycemia, tachycardia, and excessive sweating suggested possible concomitant dysautonomia.

The child started Nusinersen at age 13 days. There was a mild gradual improvement approximately 2 months after starting treatment, after the third dose, with increased small distal movements. A minimal improvement in respiratory drive was also observed, with some attempts of spontaneous breathing while receiving invasive ventilatory support. A second EET CPAP trial was then performed. The infant showed an improved respiratory function respect to the first attempt, since he increased spontaneous respiratory rate up to 61 breaths/min with mean values of Tidal Volume and spontaneous expiratory minute ventilation of 2.5 mL/kg and 122 mL/min/kg, respectively. The ETT CPAP trial had to be stopped after 55 min (as opposed to the 5 min of the first attempt) because of severe bradycardia, hyperglycemia, profuse sweating, and systemic hypertension. The transcutaneous blood gases monitoring showed increasing TcPCO2 values (81 mmHg) despite normal oxygenation.

At the age of 3 months the family opted for tracheostomy and gastrostomy. The assessments performed after 3 months showed some further increase in distal movements in both upper and lower limbs. CHOP INTEND scores went from 0 to 9.

Cardiac function progressively deteriorated with hypertrophy of the right ventricle, dilation of the right atrium, and decreased filling the left ventricle. At the age of 4 months the infant developed systemic arterial hypertension, treated with captopril. There was no sign of pulmonary hypertension.

There were also recurrent episodes of ventilator‐associated pneumonia, promptly treated with targeted antibiotic therapy. At the age of 5 months the infant died after a sudden cardiac arrest.

## Discussion

We report a case of type 0 SMA with one SMN2 copy, treated with Nusinersen. The clinical course following treatment with Nusinersen revealed some mild improvement, generally not observed in type 0.^3^ The child had no respiratory effort since birth and an attempt to switch to noninvasive ventilation around the time when treatment was started failed within a few minutes because of very severe bradycardia that prompted to return to the original settings. After treatment the child was able to tolerate a longer time on ETT CPAP trial before developing severe bradycardia. Our findings suggest that despite treatment may have produced some minimal improvement on mobility and, to some extent on respiratory function, the effect was not enough to contrast the overall severity and the multisystemic involvement. Bradycardia appeared to be the most limiting factor in considering noninvasive ventilation. Despite the choice of the family to opt for an interventional approach, including tracheostomy, recurrent infections, and a deterioration of cardiac function resulted in a cardiac arrest at the age of 5 months.

The choice of starting Nusinersen in our case was very controversial and required the involvement of our ethics board. In Italy all patients with a genetic diagnosis of 5q SMA are given the opportunity to start treatment with Nusinersen. The family was informed of the lack of data on the efficacy of Nusinersen in type 0 patients and that, following the poor efficacy found in the cases of type 1 with neonatal respiratory problems,^5^ the clinicians felt that it was unlikely to see a significant effect in a type 0 patient. Our opinion was also based on a recent consensus paper based on expert opinion, suggesting that treatment should not be started in symptomatic type 0 patients.^8^

At the time the child was born there was only one paper reporting the use of Nusinersen in a milder type 0 case who however had 2 SMN2 copies. The child had minimal improvements that did not prevent the need for tracheostomy at 8 months.^9^

The lack of published data left room for hope not only for the families but also for our ethic board who felt that a possible improvement with prolonged survival could not be excluded a priori. Since we submitted our paper, another case of a type 0 infant with 1 SMN2 copy has become available.^10^ The child had a less severe motor phenotype then ours (CHOP INTEND score of 14 as opposed to our case who had a score of 0) and was treated with both Nusinersen and Onasemnogene Abeparvovec. There was some motor improvement but this was associated with significant medical morbidity and the need for tracheostomy. Both our findings and the two published case studies will hopefully be of help for families and clinicians with a new diagnosis of type 0 at the time of considering the opportunity of treatment.

## Conflict of Interest

MP, has been in advisory boards for Roche, Biogen, and Avexis. EM is OPI and part of advisory board for Roche, Biogen Scholar Rock, and Avexis. FDT has been in advisory boards for Roche, Biogen, and Avexis. Their institution has received funding from Biogen to support a disease registry (iSMAR).
